# Co-delivery of 5-fluorouracil and miRNA-34a mimics by host-guest self-assembly nanocarriers for efficacious targeted therapy in colorectal cancer patient-derived tumor xenografts

**DOI:** 10.7150/thno.52076

**Published:** 2021-01-01

**Authors:** Jianbin Xu, Guolin Zhang, Xin Luo, Di Wang, Wei Zhou, Yan Zhang, Wei Zhang, Jiaxin Chen, Qing Meng, Engeng Chen, Heng Chen, Zhangfa Song

**Affiliations:** 1 Department of Colorectal Surgery, Sir Run Run Shaw Hospital, School of Medicine, Zhejiang University, Hangzhou, Zhejiang, 310016, P.R. China.; 2 State Key Laboratory of Medicinal Chemical Biology, Nankai University, Tianjin, 300350, P.R. China.; 3 Shenzhen Key Laboratory of Polymer Science and Technology, Guangdong Research Center for Interfacial Engineering of Functional Materials, College of Materials Science and Engineering, Shenzhen University, Shenzhen 518060, P.R. China.

**Keywords:** co-delivery, PDX model, targeted therapy, synergistic therapy

## Abstract

**Rationale:** A co-delivery system that can transport chemotherapeutic drugs and nucleotide drugs to distinct targets in tumors is an attractive strategy for cancer therapy. In this study, well-defined targeted quantum dot (QD)-based multifunctional nanocarriers were developed through self-assembly driven by host-guest interactions. 5-fluorouracil (5-FU) and microRNA-34a mimics (miR-34a(m)) were co-administered to achieve synergistic effects for colorectal cancer (CRC) therapy for the first time. Furthermore, the CRC patient-derived tumor xenograft (PDX) model, which closely mimics human CRC tumor pathological properties, was used for evaluating the therapeutic effect in this research.

**Methods:** Multiple β-cyclodextrin (CD)-attached QD nanoparticles were used as host molecules. An adamantane (ADA)-modified TCP1 peptide-targeting ligand (TCP1) was used as the guest molecule. 5-FU and miR-34a(m) were loaded into TCP1-CD-QD nanocarriers, which were used to treat CRC *in vitro* and *in vivo*. In addition, the CRC PDX model was used to evaluate the treatment efficacy of this co-delivery system.

**Results:** 5-FU and miR-34a(m) can be efficiently encapsulated into TCP1-CD-QD nanocarriers and delivered into CRC cells, which led to the inhibition of the proliferation and migration of CRC cells *in vitro* and suppression of tumor growth in a CRC cell-derived tumor xenograft model. The obtained data further suggested that co-delivery of 5-FU and miR-34a(m) could achieve synergistic effects for CRC therapy. Notably, targeted therapy via the co-delivery of 5-FU and miR-34a(m) by TCP1-CD-QD nanocarriers significantly inhibited the growth of PDX tumors.

**Conclusions:** These studies strongly indicate that such a nanocarrier-based co-delivery system is a promising combined therapeutic strategy that utilizes chemotherapeutic drugs and nucleotide drugs for enhancing colorectal cancer targeting and synergistic therapy.

## Introduction

Traditional strategies affect tumors and normal tissues simultaneously, leading to poor therapeutic effects 1. Therefore, the research and application of the tumor-targeted delivery of drugs have always been the focus of cancer therapy research 2. Nanocarrier-based drug delivery systems are of interest to the field of tumor treatment 3. Quantum dot (QD) nanoparticles have attracted increasing attention in this field due to their good biocompatibility, utility in biological imaging, and massive surface functional groups 4. In recent years, a variety of QD-based composite systems have been developed for biological imaging and diagnosis 5. Modified QDs have also been used to control drug release and gene transfection 6. β-Cyclodextrin (CD) is a class of cyclic oligosaccharides with molecular hydrophobic cavities 7. In our research, CD was used to modify the surface of QDs due to the features of CD, such as its good bioavailability and host-guest interactions 8. Then, an adamantane (ADA)-modified TCP1 peptide, as a targeting ligand, can bind to CD through host-guest interactions 9. The TCP1 peptide can specifically target the vasculature of CRC patients and CRC cells, and it can also help attach negatively charged nucleotide drugs due to the positive charge of the modified peptide 10.

5-FU is a standard chemotherapeutic drug for colorectal cancer (CRC) and can also be loaded into CD as a guest molecule to form inclusion complexes 11. However, patients show tumor recurrence after 5-FU treatment that is mainly caused by drug-resistant cancer cells 12. Therefore, enhancement of the drug sensitivity and prevention of the drug resistance of 5-FU still needs to be explored 13. MicroRNA-34a (miR-34a(m)) is a well-defined tumor suppressor of many tumor types and has been recognized as a key regulator of tumor progression 14. Previous studies showed that the expression of miR-34a not only being evaluated as a diagnostic and prognostic biomarker, but also can inhibit the growth and metastasis in many tumors 15, 16. With increasing awareness of the potential role of miR-34a in cancer therapy, some efforts are being made to delivery of miR-34a for tumor therapeutic applications. Previous studies have evaluated the effective and safe delivery of synthetic miR-34a analogs by nanocarriers and the obtained data suggest that co-delivery of chemotherapeutic agents and miR-34a could achieve treatment effects on tumor suppression 17, 18. Different from the previous studies, recently researches have shown the regulating effect of miR-34a in the response to chemotherapeutic drug 5-FU treatment. In human CRC cells, dysregulation of miR-34a expression causes drug-resistance to 5-FU 19. Other studies indicated that patients with high miR-34a expression levels benefit from 5-FU-based treatment than those with low miR-34a expression levels. It demonstrated that the combination of miR-34a and 5-FU can significantly inhibit cell growth compared to miR-34a or 5-FU alone in CRC cells 20. Therefore, the therapeutic approach to alter miRNA-34a expression has great potential to regulate the drug resistance of 5-FU to create synergetic effects. In our research, TCP1-CD-QD nanocarriers were not only loaded with 5-FU based on host-guest interactions but also loaded with miR-34a(m) due to the positive charge of the modified TCP1 peptide.

The patient-derived tumor xenograft (PDX) model is a tumor model constructed by directly transplanting tumor specimens from patients into immunodeficient mice 21. Previous studies have shown that the established PDX model retains the histological and genetic characteristics of tumor patients and maintains tumor heterogeneity 22. The subcultured tumor tissue can maintain a high degree of consistency with the initial tumor tissue, which provides good conditions for the treatment of patient cancers. It can not only help scientists' better study tumor heterogeneity and genetic complexity but also explore the possible response of patients to therapeutic agents, especially the therapeutic effect 23. Compared with the traditional animal model of tumor cell xenotransplantation, the PDX model is more accurate in evaluating the clinical application potential of some therapeutic methods, which can better reflect the clinical response 24. Using patient-derived colorectal cancer xenografts (CRC PDX), most previous studies demonstrated the mechanisms and therapeutic implications of small molecule drugs overcome resistance, immunotherapy, and regulation of tumor microenvironment 25. However, few studies have used the PDX model to evaluate the anticancer effect of nanocarrier-based therapeutic systems.

Here, PDX models from CRC patients were used to evaluate the therapeutic effect of our co-delivery system. First, we developed host-guest self-assembling TCP1-CD-QD nanocarriers for targeting CRC by using a combination therapy based on the synergistic effect of chemotherapeutic drugs and nucleotide drugs. We evaluated the therapeutic efficacy of the TCP1-CD-QD nanocarrier loaded with 5-FU and miR-34a(m) against CRC *in vitro* in a CRC cell-derived tumor xenograft model and in PDX models. In addition, we explored the potential mechanism of 5-FU and miR-34a(m) in synergistic therapy against CRC with TCP1-CD-QD nanocarriers.

## Materials and Methods

### Synthesis of CD-modified QDs

The cadmium telluride (CdTe) QDs were synthesized as previously reported 26. A zinc sulfide (ZnS) shell was added to the CdTe core with 3-mercaptopropyl acid (MPA) as the stabilizer and sulfur source (CdTe/ZnS QDs) 27. CD-functionalized QDs were prepared by covalent linking with 3-aminophenyl boronic acid (APBA) as a bridge and were synthesized as previously reported 28. In short, EDC and sulpho-NHS were added into the MPA-QD solution to activate the carboxyl groups of CdTe/ZnS QDs. Then, APBA solution was added to form an amide bond between CdTe/ZnS QDs and APBA. Amino-β-CD was added into the reaction system with stirring overnight to yield CD-modified CdTe/ZnS QDs (CD-QDs) 29.

### Synthesis of the ADA-modified TCP1 peptide

ADA-modified polyethylene glycol (PEG) was prepared by a typical reaction between amino and carbonyl chloride. Briefly, mono-amino-terminated maleimide-functionalized PEG and 1-ADA chloride were added into chloroform. After the reaction, the solvent was filtered to remove the insoluble reactants. The TCP1 peptide was introduced by click chemistry of the mercapto group and maleimide. The mixture was removed and dissolved in PBS solution. The reaction mixture was stirred at room temperature. After the reaction, the solution was dialyzed against water and lyophilized (ADA-PEG-TCP1).

### Self-assembly of TCP1-CD-QDs

CD-QDs were dissolved in PBS buffer. The ADA-PEG-TCP1 solution was added to the above solution under ultrasonic conditions for 5 min to yield the final CD-QD solution with TCP1 ligand coverage of 20 mol %. The mixture was stirred at room temperature for 30 min. Finally, the TCP1 peptide-modified CD-QDs were obtained by centrifugation at 8000 rpm and washed with H_2_O/isopropanol (v/v=1:5) three times. The TCP1-CD-QDs were resuspended by ultrasound in PBS to the desired concentrations.

### Characterization of TCP1-CD-QDs

TCP1-CD-QD morphology and size were imaged by transmission electron microscopy (TEM) using a JEM-3000F microscope (JEOL, Tokyo, Japan). The samples were prepared by placing a drop of solution onto a carbon-copper grid and air-drying for 10 min. The dynamic light scattering (DLS) was measured by a commercial LLS spectrometer (ALV/DLS/SLS-5022F) equipped with a multidigital time correlator and a cylindrical laser. Nuclear magnetic resonance (NMR) was employed to monitor the synthesized functional molecules.

### Cell culture

Human CRC cell line DLD1 (ATCC® CCL‑221^TM^) cells were purchased from the American Type Culture Collection (ATCC, USA). Cell lines from passage 10 were used in all experiments. Cells were cultured in Dulbecco's modified Eagle medium (DMEM), which contained 10% fetal bovine serum (FBS), penicillin (10 U/mL) and streptomycin (10 mg/mL), under a humid environment with 5% (v/v) CO_2_ and 95% air at 37 °C.

### Cytotoxicity of TCP1-CD-QDs

DLD1 cells were seeded into a 96-well plate (1 × 10^4^ cells/well) and incubated for 24 h at 37 °C. The media was then replaced with a suspension of QDs or TCP1-CD-QDs at various concentrations in culture medium, followed by incubation for 48 h. The cells were treated with a mixture of fresh culture medium/CCK-8 solution (200 mL/20 mL, v/v) in each well. After incubation for another 4 h, the optical density of the mixture was measured by a microplate reader (Bio-Rad 680, USA) at a wavelength of 450 nm.

### Cellular uptake of TCP1-CD-QDs

The cellular uptake behavior and the intracellular distribution of the TCP1-CD-QDs were analyzed using both flow cytometry and confocal laser scanning microscopy (CLSM). For flow cytometry analysis, DLD1 cells were seeded onto a 6-well plate (2 × 10^5^ cells/well) and cultured for 24 h. The cells were treated with QDs and TCP1-CD-QDs (concentration: 20 nM) for 4 h. The treated cells were then washed 3 times with PBS, trypsinized, centrifuged, and resuspended in PBS. Finally, the analysis was performed using a flow cytometer (Beckman, California, USA). For CLSM analysis, DLD1 cells were seeded in a 24-well plate (5 × 10^4^ cells/well) and cultured for 24 h to allow cell attachment. The cells were treated with TCP1-CD-QDs for 4 h (concentration: 20 nM) and fixed with paraformaldehyde for 15 min. The cells were treated with 4',6-diamidino-2-phenylindole (DAPI) for 15 min for nuclear staining and washed 3 times with PBS. Finally, the cells were imaged using a Nikon Digital Eclipse C1si confocal laser scanning microscope (Nikon, Tokyo, Japan).

### Loading of 5-FU and miR-34a(m) into TCP1-CD-QDs

5-FU was loaded into the TCP1-CD-QDs through host-guest interactions with CD as previously reported 30. Specific amounts of 5-FU and TCP1-CD-QDs were mixed together and sonicated for 5 min and then stirred overnight to form the TCP1-CD-QDs/5-FU complexes. After centrifugation to collect the complexes, the supernatant was collected to test the unloaded 5-FU by UV-vis spectrophotometry (UV-2600, Japan) at a wavelength of 266 nm. The loading capacity (LC) and encapsulation efficiency (EE) of 5-FU were determined according the following Eqs. (1) and (2). 5-FU release experiments were performed in PBS at 37 °C using a dialysis method 31. TCP1-CD-QDs/5-FU was placed in a dialysis membrane bag and then immersed in PBS and stirred. Free 5-FU was used as a control. At specific time intervals, 5 mL of PBS was collected, and an equal volume of PBS was added. The binding of miR-34a(m) to the TCP1-CD-QDs was performed at various molar ratios and detected by agarose gel electrophoresis. The miRNA-34a mimic sequences used in our study were sense-UUCUCCGAACGUGUCACGUdTdT and antisense-ACGUGACACGUUCGGAGAAdTdT.

LC (wt%) = (weight of loaded 5-FU/weight of nanocarrier) × 100% (1)

EE (wt%) = (weight of loaded 5-FU/weight of 5-FU in feed) × 100% (2)

### Cell proliferation

DLD1 cell proliferation was investigated through a CCK-8 assay. The cells were seeded into a 96-well plate (1 × 10^4^ cells/well) and incubated for 24 h at 37 °C. The media was then replaced with PBS, 5-FU, TCP1-CD-QDs/5-FU, TCP1-CD-QDs/miR-34a(m), and TCP1-CD-QDs/5-FU-miR-34a(m) at a 5-FU concentration of 2 μM and a miR-34a(m) concentration of 25 nM in culture medium, followed by incubation for 48 h. Untreated DLD1 cells were used as a control. Then, the cells were treated with a mixture of fresh culture medium/CCK-8 solution (200 mL/20 mL, v/v) per well. After incubation for another 4 h, the optical density of the mixture was measured by a microplate reader (Bio-Rad 680, USA) at a wavelength of 450 nm.

### Cell migration

DLD1 cell migration was investigated using a Transwell assay. The bottom chambers of the Transwell plate (Millipore, USA) were filled with medium with FBS, and the top chambers of the Transwell plate were seeded with medium without FBS containing 1 × 10^4^ DLD1 cells. Then, PBS, 5-FU, TCP1-CD-QDs/5-FU, TCP1-CD-QDs/miR-34a(m) and TCP1-CD-QDs/5-FU-miR-34a(m) were added to the top chambers. DLD1 cells were allowed to migrate for 24 h, and then the nonmigrated cells were removed. The migrated cells were fixed with 100% methanol and stained with 0.05% crystal violet. The migration of DLD1 cells was also evaluated by wound healing assays. PBS, 5-FU, TCP1-CD-QDs/5-FU, TCP1-CD-QDs/miR-34a(m) and TCP1-CD-QDs/5-FU-miR-34a(m) were supplemented with culture medium. The migration was quantified by area calculations, and the percentage of migration was calculated using PBS as a control.

### Gene expression analysis

DLD1 cells were treated with PBS, 5-FU, TCP1-CD-QDs/5-FU, TCP1-CD-QDs/miR-34a(m) and TCP1-CD-QDs/5-FU-miR-34a(m) of the 5-FU concentration at 2 μM and the miR-34a(m) concentration of 25 nM in culture medium, followed by incubation for 48 h, then the RNA were extracted. An equal amount of RNA from each sample was reverse-transcribed into cDNA using reverse transcriptase and oligoDT. Real time PCR was performed on an Applied Biosystems 7300 Real-Time PCR system. The relative gene expression was calculated using the ΔΔCT method, where fold difference was calculated using the expression 2ΔΔCt. β-actin was used as an internal control.

### Western blotting analysis

For Western blotting analysis, DLD1 cells were treated with PBS, 5-FU, TCP1-CD-QDs/5-FU, TCP1-CD-QDs/miR-34a(m) and TCP1-CD-QDs/5-FU-miR-34a(m) of the 5-FU concentration at 2 μM and the miR-34a(m) concentration of 25 nM in culture medium, followed by incubation for 48 h, then the cell proteins were extracted. An equal amount of protein was separated on the SDS-PAGE, transferred onto nitro-cellulose membrane, and blocked and incubated overnight with monoclonal antibodies against Sirt1 (1:1000), p53 (1:2000), CD44 (1:2000) and β-actin (1:2000) overnight at 4 °C. After washing, the membrane was incubated with HRP-conjugated secondary antibody (1:5000) for 2 h at room temperature. The bands were visualized using the Westzol enhanced chemiluminescence kit (Intron, Sungnam, Korea) and the expression was normalized with housekeeping gene expression. The chemiluminescence signals were detected with a chemiluminescent ECL substrate (Millipore) under an imaging system (Bio-Rad, Hercules, CA, USA).

### *In vivo* studies

All animal studies were approved by the Zhejiang University Animal Care and Use Committee and performed in accordance with national guidelines and regulations. A human CRC cell-derived tumor xenograft model was established by subcutaneously injecting DLD1 cells into the left flank of female nude mice. When the tumor volume reached ~100 mm^3^, mice were intravenously administered PBS, 5-FU, TCP1-CD-QDs/5-FU, TCP1-CD-QDs/miR-34a(m) or TCP1-CD-QDs/5-FU-miR-34a(m). The treatment was given every 4 days for 4 weeks. The tumor volumes were measured and calculated every 4 days according to π/6 (width^2^ × length). The animals were then sacrificed and tumor tissues and major organs were collected for further analysis.

### PDX model studies

Fresh colorectal tumor tissue was obtained from consenting patients at Sir Run Run Shaw Hospital, School of Medicine, Zhejiang University; the study was approved by the local institutional Ethics Committee (institutional review board reference no. 20140213-19). All patients provided written consent to allow their tissue to be stored and used for research, and all specimens were deidentified and not linked with any personal health information. Tumor specimens were cut into 2 mm^3^ pieces and placed in the left flank of female nude mice. When the tumor volume reached ~100 mm^3^, mice were intravenously administered PBS, 5-FU, TCP1-CD-QDs/5-FU, TCP1-CD-QDs/miR-34a(m) or TCP1-CD-QDs/5-FU-miR-34a(m). The treatment was given every 4 days for 2-3 weeks. The tumor volumes were measured and calculated every 4 days according to π/6 (width^2^ × length). The animals were then sacrificed, and tumor tissues were collected for further analysis.

### Histological analysis

The collected tissues were fixed in 10% formalin and embedded in paraffin. The tissue sections were then deparaffinized in xylene and rehydrated in graded ethanol. Standard staining with hematoxylin and eosin (H&E) and immunohistochemical (IHC) staining were performed. The tissue sections were then observed under a microscope.

### Statistical analysis

All data are presented as the mean ± standard deviation. Statistical analysis was performed by two-way ANOVA and Tukey's HSD post hoc test to allow for comparison between groups, with the experimental group as the independent factor. *p < 0.05, **p < 0.01, ***p < 0.001, ^#^*p* < 0.05, ^##^*p* < 0.01, ^###^*p* < 0.001.

## Results and Discussion

### Synthesis and characterization of TCP1-CD-QD nanocarriers

**Figure 1:**
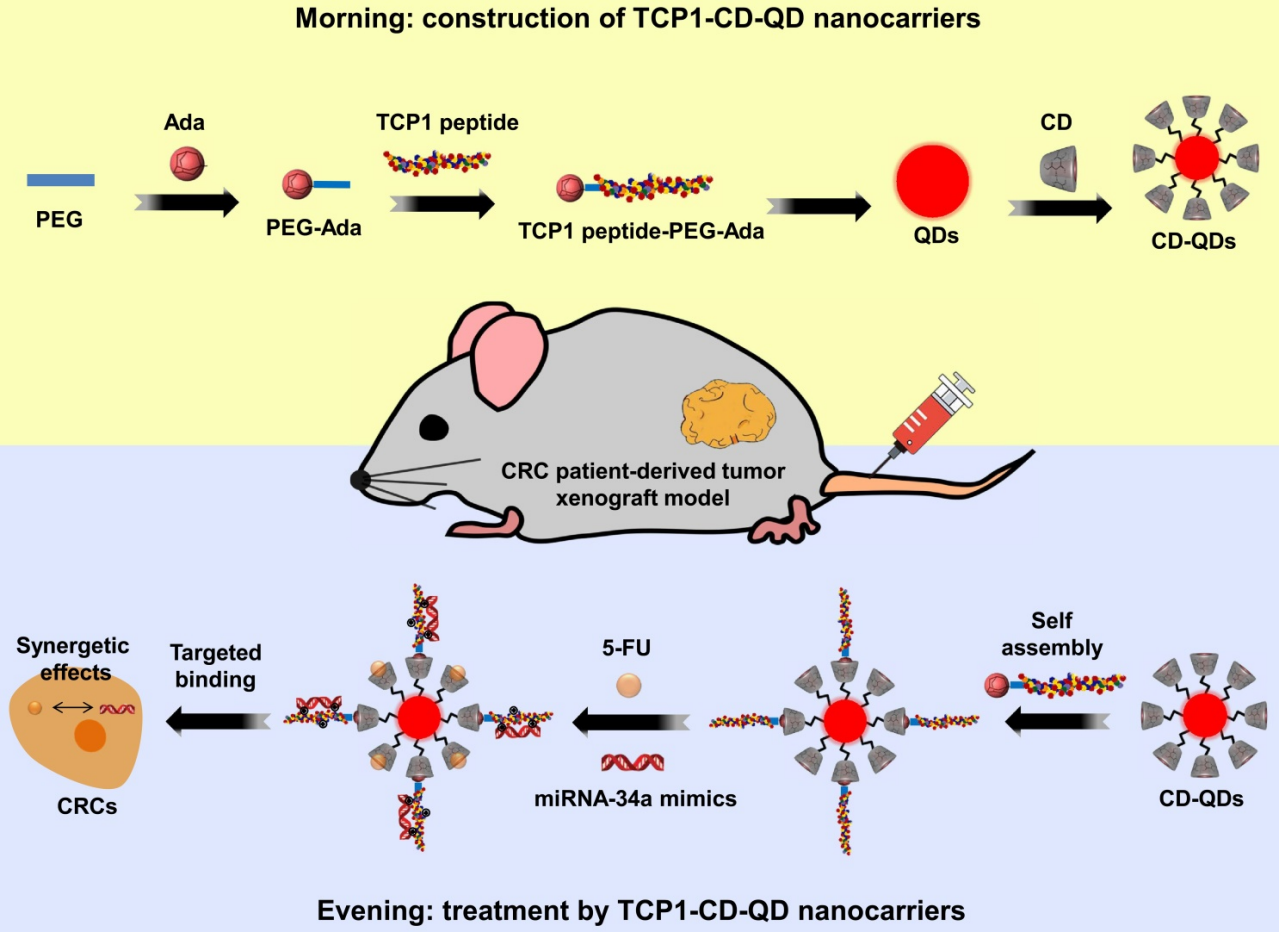
Schematic illustration of the fabrication of host-guest self-assembled nanocarriers using CD-functionalized QDs (CD-QDs) and the ADA-functionalized targeting peptide (TCP1 peptide-PEG-ADA) loaded with chemotherapeutic drugs (5-FU, 5-fluorouracil) and nucleotide drugs (miRNA-34a mimics) for synergistic therapy of CRC.

The targeted TCP1-CD-QD nanocarriers were designed to form by self-assembly of CD-conjugated QDs (CD-QDs) and ADA-functionalized peptides (ADA-PEG-TCP1) via host-guest interactions (**Figure 1**). The binding of CD and CdTe/ZnS QDs was confirmed by ^1^H NMR spectrum (**Figure S1**). CD and ADA complexes have been used in various supramolecular systems for biomedical applications 32, 33. Therefore, ADA-PEG-TCP1 was designed to incorporate the targeting probe into the CD-QD surface. ADA-PEG was prepared by a typical reaction between amino groups and carbonyl chloride 34. The TCP1 peptide was introduced by click chemistry of the mercapto group and maleimide 35. The ^1^H NMR spectrum showed that the TCP1 peptide on ADA-PEG was successfully modified (**Figure S2**). Then, the TCP1 peptide (ADA-PEG-TCP1) bound to the surface of CD-QDs via the host-guest interaction between ADA and CD 36.

**Figure 2:**
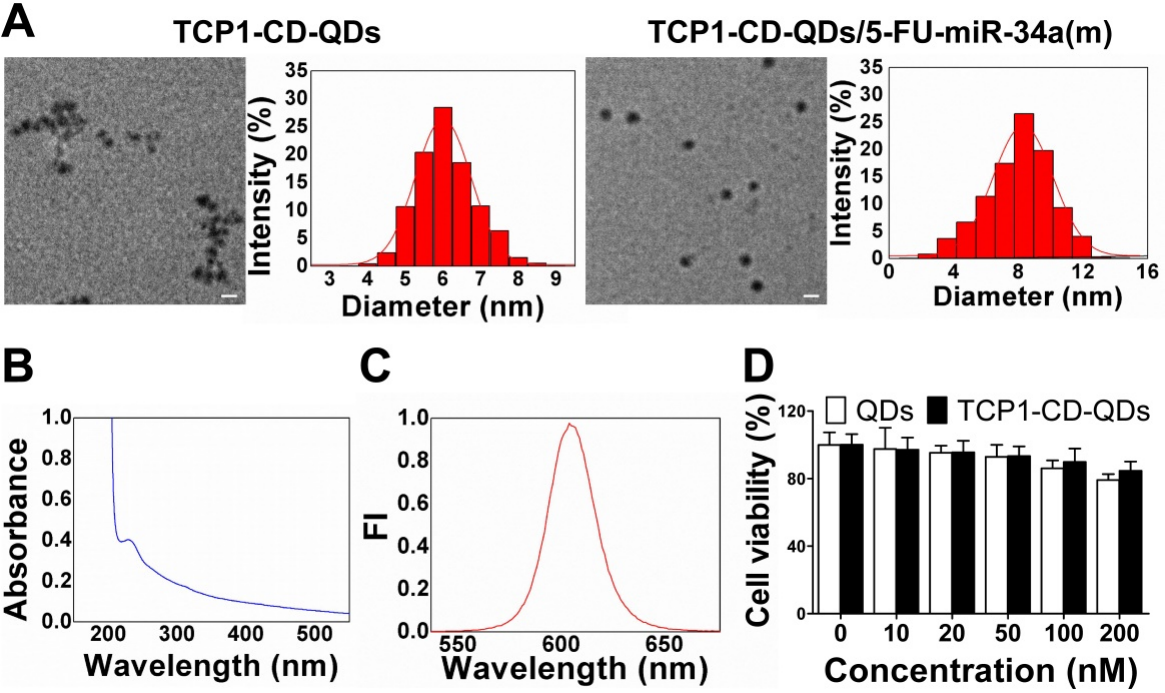
** Representative characterizations of TCP1-CD-QD nanocarriers. (A)** TEM images and DLS analysis of TCP1-CD-QDs and TCP1-CD-QDs/5-FU-miR-34a (m). Bar: 10 nm. **(B)** UV-vis spectra of TCP1-CD-QD nanocarriers. **(C)** Fluorescence intensity of the TCP1-CD-QD nanocarriers. **(D)** Cell cytotoxicity of TCP1-CD-QD nanocarriers toward DLD1 cells after 48 h.

The morphology of the TCP1-CD-QD nanocarriers and TCP1-CD-QD nanocarriers loading with 5-FU-miR-34a(m) were elucidated by TEM and DLS. **Figure 2A** shows representative images and DLS results of the prepared TCP1-CD-QDs and TCP1-CD-QDs/5-FU-miR-34a(m). The mean diameter of the TCP1-CD-QDs was approximately 5-7 nm, which is consistent with the results obtained from DLS. After the loading of 5-FU and miR-34a(m), the morphology and size of TCP1-CD-QDs has a slight increased to 7-9 nm. As shown in **Table S1**, compared to the TCP1-CD-QDs, the zeta potential of the TCP1-CD-QDs/5-FU-miR-34a(m) nanocomplexes significantly decreases, indicating the successful uploading of the miR-34a(m). This result showed that the TCP1-CD-QDs and TCP1-CD-QDs/5-FU-miR-34a(m) were well dispersed and uniform in shape and size. To evaluate the stability of nanocarriers, TCP1-CD-QDs and TCP1-CD-QDs/5-FU-miR-34a(m) were scattered in PBS and serum solution at 37 °C for 72 h. As shown in **Figure S3,** the mean sizes of TCP1-CD-QDs and TCP1-CD-QDs/5-FU-miR-34a(m) tested by DLS varied little in PBS or serum solution. The results suggested that the TCP1-CD-QDs and TCP1-CD-QDs/5-FU-miR-34a(m) were stable in the salt and serum solution. Furthermore, the TCP1-CD-QD nanocarriers have excellent fluorescence properties, as evidenced by a wide UV excitation spectrum (**Figure 2B**) and narrow emission band (~610 nm) (**Figure 2C**), which is essential to the labeling of cancer cells 37. For cytotoxicity analysis, **Figure 2D** shows that over 84% of DLD1 cells were viable even after incubation for 48 h in the presence of TCP1-CD-QD nanocarrier concentrations as high as 200 nM.

### Cellular uptake and drug co-delivery of TCP1-CD-QD nanocarriers

**Figure 3:**
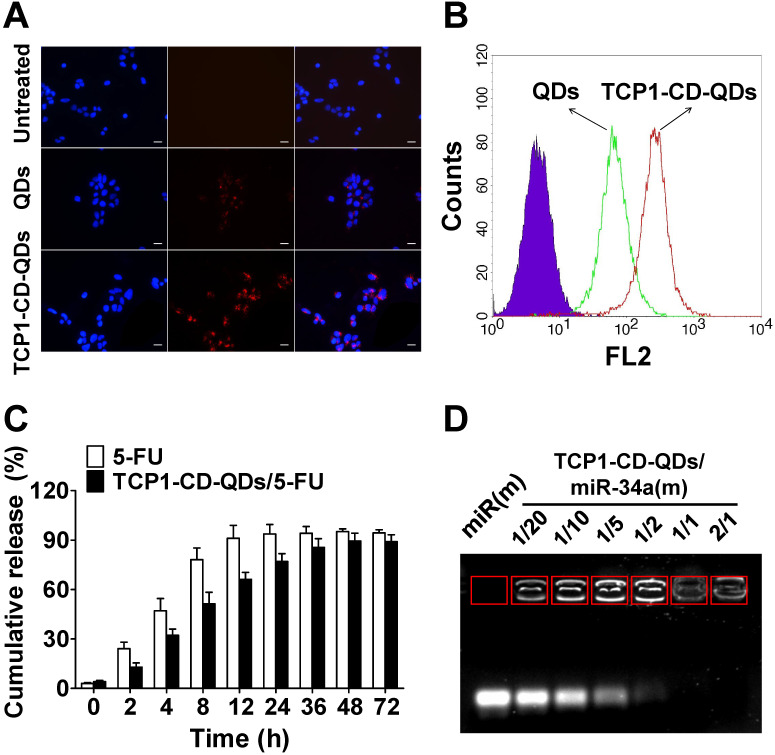
** Cellular uptake and drug co-delivery of TCP1-CD-QD nanocarriers. (A)** Cells were incubated with QD (20 nM) and TCP1-CD-QD (20 nM) nanocarriers for 4 h. Cell nuclei were stained with DAPI. Bar: 10 µm. **(B)** Flow cytometry analysis of the endocytosis of QDs (green peak) and TCP1-CD-QDs (red peak) in DLD1 cells for 4 h. **(C)** Drug release profiles of free 5-FU and TCP1-CD-QDs/5-FU nanocomplexes in PBS solution. **(D)** Effective binding of miR-34a(m) onto the TCP1-CD-QD nanocarrier as determined by agarose gel electrophoresis. Increasing the concentration of TCP1-CD-QDs reduces the mobility of miR-34a(m) during electrophoresis, thus indicating the formation of TCP1-CD-QDs/miR-34a(m) nanocomplexes.

For the cellular uptake studies, CLSM and flow cytometry were used to evaluate the effect of the TCP1 peptide on cellular uptake in CRC cells. As shown in **Figure 3A**, CLSM was used to visualize TCP1-CD-QD nanocarriers in the cells to determine the intracellular distribution. As predicted, red fluorescence was observed in DLD1 cells incubated with the nanocarriers. The endocytosis efficiency of QDs or TCP1-CD-QD nanocarriers in DLD1 cells was analyzed by flow cytometry (**Figure 3B**). DLD1 cells treated with TCP1-CD-QD nanocarriers showed higher fluorescence levels than those treated with QDs. DLD1 cells without any treatment were used as a negative control. Due to the specific targeting of CRC cells and the vasculature of orthotropic CRC patients by TCP1 peptide, the TCP1 peptide has been used to modify drug carriers for targeted CRC therapy 38. These results confirm that the TCP1-CD-QD nanocarriers enhanced CRC cell internalization, which is essential for targeted drug delivery.

### Co-delivery of 5-FU and miR-34a(m) by TCP1-CD-QD nanocarriers

The property of drug release from the co-delivery system is very important for anticancer efficacy 39. For an ideal drug delivery system, it is essential to protect the drug from degradation in the biological environment 40, 41. 5-FU is an effective chemotherapeutic drug for treating a variety of cancers, including liver cancer, breast cancer and CRC 42. 5-FU can be effectively loaded into TCP1-CD-QD nanocarriers by self-assembly with CD 43. First, we found that the LC and EE of 5-FU by TCP1-CD-QD nanocarriers were approximately at 6.4% and 25.8%, respectively. To investigate the 5-FU release behavior, TCP1-CD-QDs/5-FU were placed in a dialysis membrane bag and then immersed in PBS buffer (free 5-FU was used as a control). As shown in **Figure 3C**, approximately 90% of free 5-FU was released after 12 h, and approximately 60% of 5-FU showed sustained release from TCP1-CD-QD nanocarriers after 12 h. The miR-34a(m) binding ability of TCP1-CD-QDs was investigated through the agarose gel retardation assay. As shown in **Figure 3D**, free miR-34a(m) migrated to the bottom and exhibited a bright band. When the molar ratio of TCP1-CD-QDs to miR-34a(m) increased (1:20 to 1:2), a weak band was observed at the bottom, indicating a small amount of free miR-34a(m) in the solution. No bright bands were observed when the molar ratio of TCP1-CD-QDs to miR-34a(m) was over 1:1. These results suggested that our TCP1-CD-QD nanocarriers can simultaneously carry 5-FU and miR-34a(m) effectively.

### Cell apoptosis and migration

**Figure 4:**
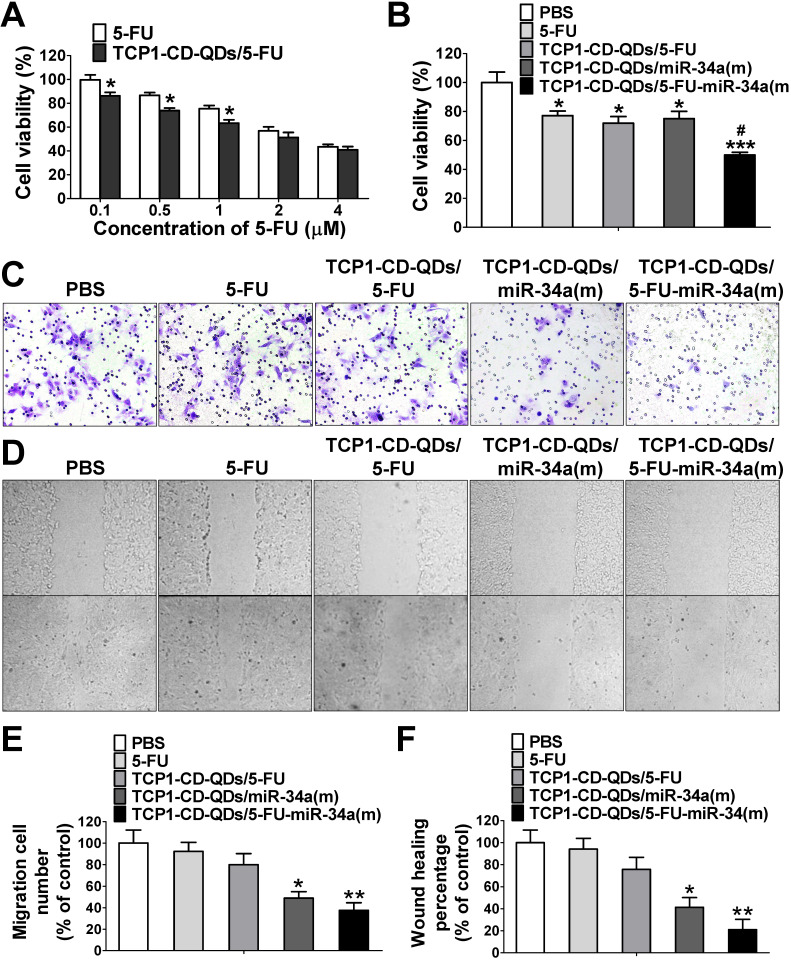
** Cell apoptosis and migration assays of TCP1-CD-QDs/5-FU-miR-34a (m). (A)** Cell viability of DLD1 cells treated with 5-Fu and TCP1-CD-QDs/5-Fu at different concentrations (concentrations of 5-FU from 0.1 to 4 µM). The cells were treated for 72 h and measured. **(B)** Cell viability of cells treated with PBS, free 5-FU, TCP1-β-CD-QDs/5-FU, TCP1-β-CD-QDs/miR-34(m) and TCP1-β-CD-QDs/5-FU+miR-34(m). The concentration of 5-FU at 2 µM and miR-34a(m) at 25 nM in all treatments for 48 h was measured. **(C, E)** Transwell assays of cells treated with PBS, free 5-FU, TCP1-CD-QDs/5-FU, TCP1-CD-QDs/miR-34(m) and TCP1-CD-QDs/5-FU+miR-34(m). **(D, F)** Wound-healing assays of cells treated with PBS, free 5-FU, TCP1-CD-QDs/5-FU, TCP1-CD-QDs/miR-34(m) and TCP1-CD-QDs/5-FU+miR-34(m). The concentration of 5-FU at 2 µM and miR-34a(m) at 25 nM in all treatments for 24 h was measured. The data are reported as the mean ± SD of the experiments (n = 3). *p < 0.05, **p < 0.01, ***p < 0.001, ^#^*p* < 0.05.

To investigate whether the TCP1-CD-QD-mediated intracellular co-delivery of 5-FU and miR-34a(m) can effectively increase the chemotherapeutic efficacy of 5-FU, the apoptotic effects of TCP1-CD-QDs/5-FU-miR-34a(m) against CRC cells after 72 h in different formulations was evaluated by CCK-8 assays. **Figure 4A** shows that increasing the 5-FU content (0.1 to 4 μM) resulted in an enhanced anticancer effect, implying that DLD1 cell inhibition was concentration-dependent, which was consistent with previous reports 44. At the same time, treatment with TCP1-CD-QDs/5-FU-miR-34a(m) resulted in a cell viability of 49.9% ± 3.2%, which was higher than that of treatment with free 5-FU (77.1% ± 5.5%), TCP1-CD-QDs/5-FU (71.9% ± 7.8%) or TCP1-CD-QDs/miR-34a(m) (75.1% ± 8.7%) (**Figure 4B**). It has been reported that miRNA-34a has synergistic anticancer effects when combined with conventional chemotherapy 45. After delivering miR‑34a(m) via TCP1-CD-QD nanocarriers into CRC cells, miR‑34a expression was significantly increased compared with that in the untreated groups (**Figure S4**). Metastasis-related tumor recurrence is still common and responsible for the majority of CRC-associated mortality 46. Previous studies showed that miRNA-34a is a key negative regulator of metastasis 47. To investigate whether TCP1-CD-QDs/5-FU-miR-34a(m) as therapeutic agents can effectively inhibit migration, a Transwell assay was performed to evaluate CRC cell migration. **Figure 4C and 4E** shows that no obvious inhibition of cell migration was observed in cells treated with PBS, free 5-FU (92.3% ± 14.6%) or TCP1-CD-QDs/5-FU (79.9% ± 17.8%). In contrast, significant inhibition of the migration of DLD1 cells was observed in cells treated with TCP1-CD-QDs/miR-34a(m) (49.0% ± 10.3%) and TCP1-CD-QDs/5-FU-miR-34a(m) (37.6% ± 12.1%) by a Transwell assay, indicating that the inhibition of cell migration is mediated by the delivery of miRNA-34a mimics. Wound healing assays support our results. As shown in **Figure 4D and 4F**, TCP1-CD-QDs/miR-34a(m) (41.3% ± 15.4%) and TCP1-CD-QDs/5-FU-miR-34a(m) (21.0% ± 16.2%) showed higher migration inhibition of DLD1 cells than PBS, free 5-FU (94.1% ± 16.9%) and TCP1-CD-QDs/5-FU (75.7% ± 19.0%). In addition, as shown in **Figure S5** and **Figure S6**, treatment with TCP1-CD-QDs/5-FU-miR-34a(m) resulted in a higher reduction of HCT116 and RKO cells viability compared to free 5-FU, TCP1-CD-QDs/5-FU or TCP1-CD-QDs/miR-34a(m). As shown in **Figure S7** and **Figure S8**, a significant inhibition of the migration of HCT116 and RKO cells was observed in cells treated with TCP1-CD-QDs/miR-34a(m) and TCP1-CD-QDs/5-FU-miR-34a(m) by a Transwell assay. It indicated that the co-delivery of 5-FU and miR-34a(m) by TCP1-CD-QDs improved the apoptotic effect and inhibited the metastasis of more than one CRC cell lines. These studies suggested that co-delivery of 5-FU and miR-34a(m) by TCP1-CD-QDs improved the apoptotic effect and inhibited the metastasis of CRC cells.

### The synergistic effect of co-delivery of 5-FU and miR-34(m) by TCP1-CD-QD nanocarriers

**Figure 5:**
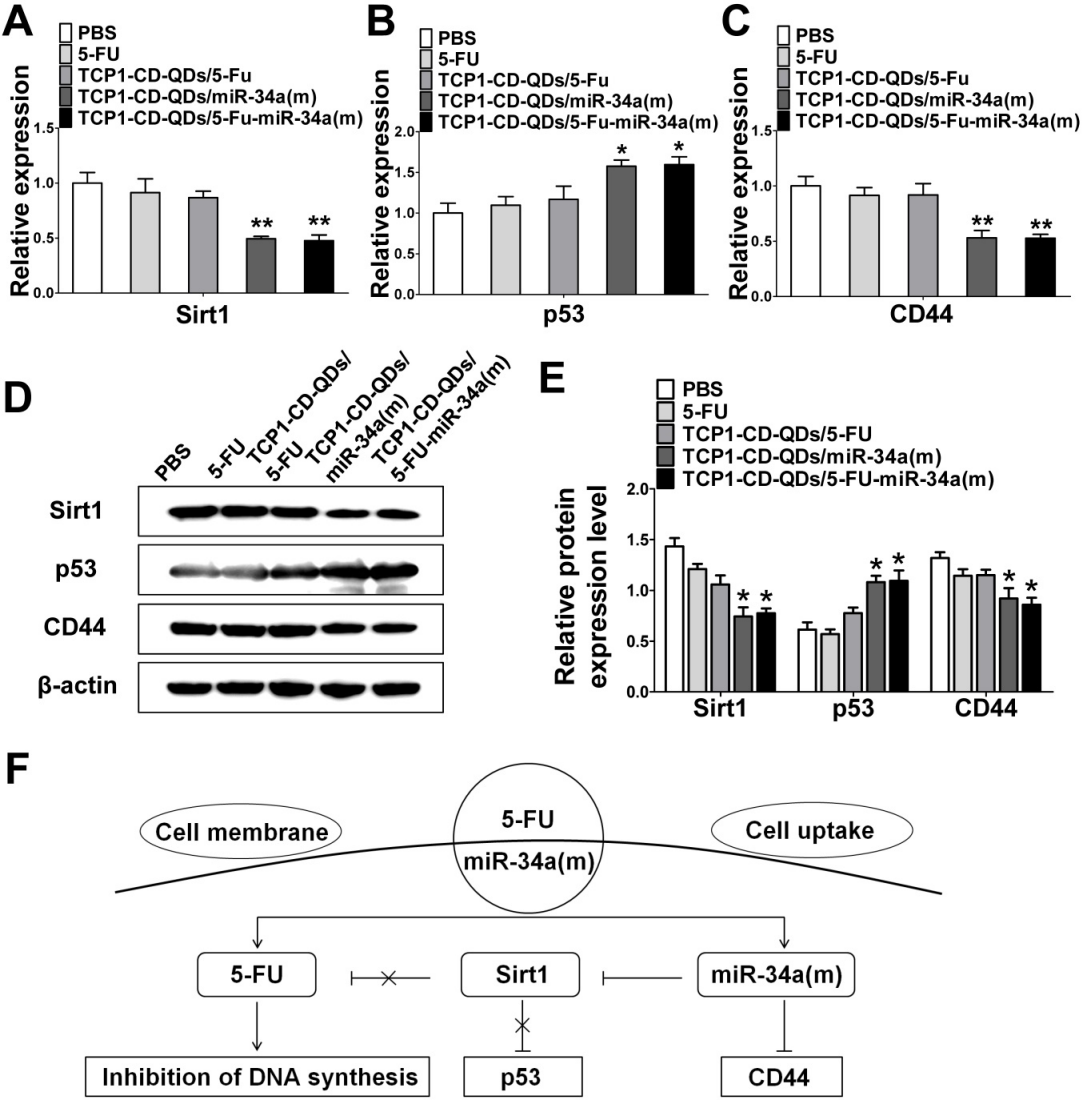
** The synergistic effect of the co-delivery of 5-FU and miR-34a(m) by TCP1-CD-QD nanocarriers. (A-C)** The mRNA expression of the expression of target proteins in DLD1 cells. **(D-E)** Western blotting and band intensities of the expression of target proteins in DLD1 cells were analyzed. The relative expression levels of Sirt1, p53 and CD44 in cells treated with PBS, free 5-FU, TCP1-CD-QDs/5-FU, TCP1-CD-QDs/miR-34a(m) and TCP1-CD-QDs/5-FU-miR-34a(m) for 48 h. β-actin was used as an internal control. **(F)** Schematic representation of the potential synergistic mechanism of the co-delivery of 5-FU and miR-34 (m) by TCP1-CD-QD nanocarriers in CRC treatment.

Next, we explored the potential mechanism involved in the improvement of apoptosis and inhibition of metastasis by co-delivery of 5-FU and miR-34(m) with TCP1-CD-QD nanocarriers. We detected the relative expression level of mRNA and protein for sirt1, p53 and CD44 by real time PCR and western blotting analysis. As shown in **Figure 5A-E**, the dramatic downregulation of sirt1 expression was observed in DLD1 cells treated with TCP1-CD-QDs/5-FU-miR-34a(m), while no significant changes were observed in the PBS, free 5-FU and TCP1-CD-QDs/5-FU groups. In 5-FU-resistant cells, the expression of miRNA-34a was sustained at a low level 48. Sirt1 is one of the target genes for miRNA-34a and is related to drug resistance 49. In particular, the silencing of sirt1 significantly reduced the resistance to 5-FU of 5-FU-resistant cells 50. Therefore, co-delivery of miRNA-34a mimics by TCP1-CD-QD nanocarriers targeting sirt1 could negatively regulate, at least in part, the resistance toward 5-FU of CRC cells. The function of p53 as a tumor suppressor has been attributed to its ability to promote cell death or permanently inhibit cell proliferation 51. In our studies, the upregulation of the p53 protein was detected in the TCP1-CD-QDs/5-FU-miR-34a(m) group. Previous studies have demonstrated that CD44 is implicated in tumor progression and metastasis 52. The expression of CD44 is increased in CRC and correlates with poor clinical outcomes 53. It has also been shown that miRNA-34a is a key negative regulator of the CD44 protein in cancer cells 54. Our studies showed a remarkable downregulation of CD44 expression in DLD1 cells treated with TCP1-CD-QDs/5-FU-miR-34a(m). In summary, our results showed a synergistic effect of the co-delivery of 5-FU and miRNA-34a mimics by TCP1-CD-QDs for CRC therapy. Compared to the PBS group, the free 5-FU, TCP1-CD-QDs/5-FU and TCP1-CD-QDs/5-FU-miR-34a(m) groups displayed more intensive anticancer activity by simultaneously inducing tumor cell death via 5-FU, reducing drug resistance and inhibiting tumor cell migration by activating apoptosis/metastasis/drug resistance-related pathways via the miRNA-34a mimic (**Figure 5F**).

### Anticancer efficacy in a CRC cell-derived tumor xenograft model

**Figure 6:**
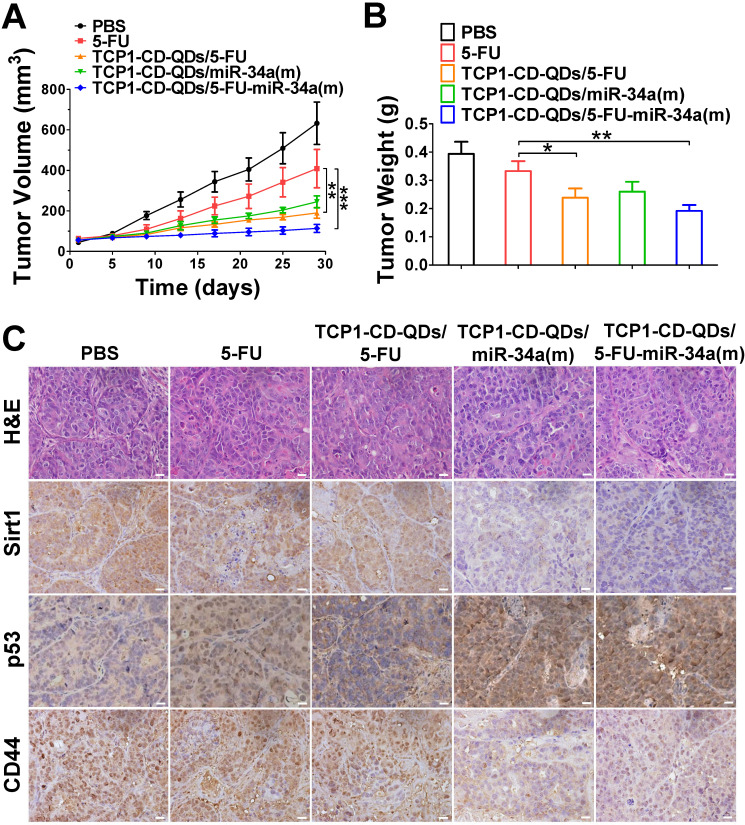
** Suppression of subcutaneous tumor growth by TCP1-CD-QDs/5-FU+miR-34(m) in tumor-bearing mice.** The tumor models were treated with PBS, free 5-FU, TCP1-CD-QDs/5-FU, TCP1-CD-QDs/miR-34a(m) and TCP1-CD-QDs/5-FU-miR-34a(m) group, respectively. **(A)** The tumor growth in subcutaneous tumor model of treatments in 5 groups. **(B)** The tumor weight in subcutaneous tumor model of treatments in 5 groups. **(C)** Representative sirt1, p53 and CD44 immunohistochemistry images of treatments in 5 groups. *p < 0.05, **p < 0.01, ***p < 0.001. Bar: 20 µm.

The anticancer efficacy of TCP1-CD-QDs/5-FU-miR-34a(m) was studied in nude mice bearing subcutaneous DLD1 tumor xenografts. As shown in **Figure 6**, the animals were treated with PBS, free 5-FU, TCP1-CD-QDs/5-FU, TCP1-CD-QDs/miR-34a(m) and TCP1-CD-QDs/5-FU-miR-34a(m). Obvious inhibition of tumor growth was observed in the free 5-FU, TCP1-CD-QDs/5-FU, TCP1-CD-QDs/miR-34a(m) and TCP1-CD-QDs/5-FU-miR-34a(m) groups compared with that in the PBS group. In particular, enhanced tumor growth inhibition efficacy was observed in the TCP1-CD-QDs/5-FU-miR-34a(m) group (**Figure 6A**). Meanwhile, the mean tumor weight of the TCP1-CD-QDs/5-FU-miR-34a(m) group was significantly lower than the weight of the control 5-FU group (**Figure 6B**). Consistent with the *in vitro* results, immunohistochemical staining confirmed that sirt1/CD44 were more highly expressed and p53 was more lowly expressed in the PBS-, free 5-FU-, and TCP1-CD-QDs/5-FU-treated groups. Conversely, sirt1/CD44 expression was decreased and p53 expression was increased in the TCP1-CD-QDs/5-FU-miR-34a(m)-treated group (**Figure 6C**). These studies suggested that TCP1-CD-QDs/5-FU-miR-34a(m) could achieve enhanced inhibition of anticancer effects in CRC cell-derived tumor xenograft models.

### Anticancer efficacy in CRC PDX models

**Figure 7:**
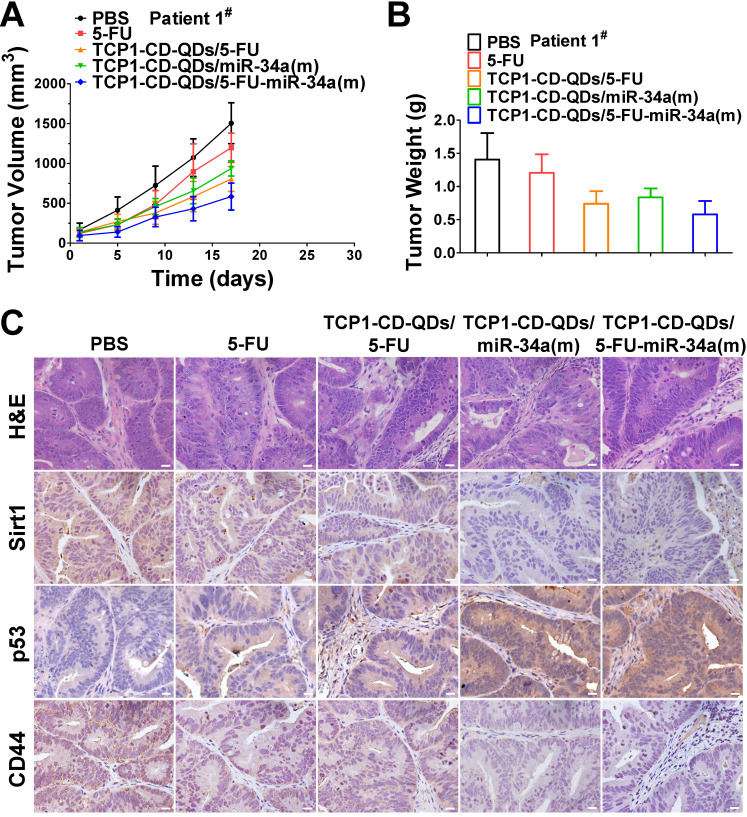
** Suppression of subcutaneous tumor growth by TCP1-CD-QDs/5-FU+miR-34(m) in PDX models.** The tumor models were treated with PBS, free 5-FU, TCP1-CD-QDs/5-FU, TCP1-CD-QDs/miR-34a(m) and TCP1-CD-QDs/5-FU-miR-34a(m) group, respectively. **(A)** The tumor growth in PDX model of treatments in 5 groups. **(B)** The tumor weight in PDX model of treatments in 5 groups. **(C)** Representative sirt1, p53 and CD44 immunohistochemistry images of treatments in 5 groups. Bar: 20 µm.

PDX models preserve the heterogeneity observed in fresh human tumor samples better than tumor xenografts derived from cell lines 55. They have become the preferred *in vivo* tool for developing strategies for anticancer nanomedicine research 56. Therefore, we analyzed the anticancer efficacy of the co-delivery of 5-FU and miR-34a(m) by TCP1-CD-QD nanocarriers in CRC PDX models. As shown in **Figure 7**, PDX models were treated with PBS, free 5-FU, TCP1-CD-QDs/5-FU, TCP1-CD-QDs/miR-34a(m) and TCP1-CD-QDs/5-FU-miR-34a(m). Our results showed that an effective reduction of the tumor volume in the TCP1-CD-QDs/5-FU-miR-34a(m) group was observed compared to that in the control groups (**Figure 7A**). As shown in **Figure 7B**, treatment with TCP1-CD-QDs/5-FU-miR-34a(m) resulted in a lower tumor weight compared to that with either TCP1-CD-QDs/5-FU or TCP1-CD-QDs/miR-34a(m). We detected the expression levels of sirt1, p53 and CD44 in tumor tissues obtained from mice treated with PBS, free 5-FU, TCP1-CD-QDs/5-FU, TCP1-CD-QDs/miR-34a(m) and TCP1-CD-QDs/5-FU-miR-34a(m) by immunohistochemical staining (**Figure 7C**). Lower expression levels of sirt1 and CD44 were detected in the TCP1-CD-QDs/miR-34a(m)-treated group and especially in the TCP1-CD-QDs/5-FU-miR-34a(m)-treated group compared to those in the control groups. Conversely, higher expression levels of p53 expression was detected in the TCP1-CD-QDs/miR-34a(m)- and TCP1-CD-QDs/5-FU-miR-34a(m)-treated group. Supporting the above results, similar results were obtained in different CRC PDX models with the same treatments (**Figure S9-10**). These studies suggested that TCP1-CD-QDs/5-FU-miR-34a(m) could achieve enhanced anticancer effects in CRC PDX models.

### TCP1-CD-QD nanocarriers reduce the systemic toxicity of drugs

**Figure 8:**
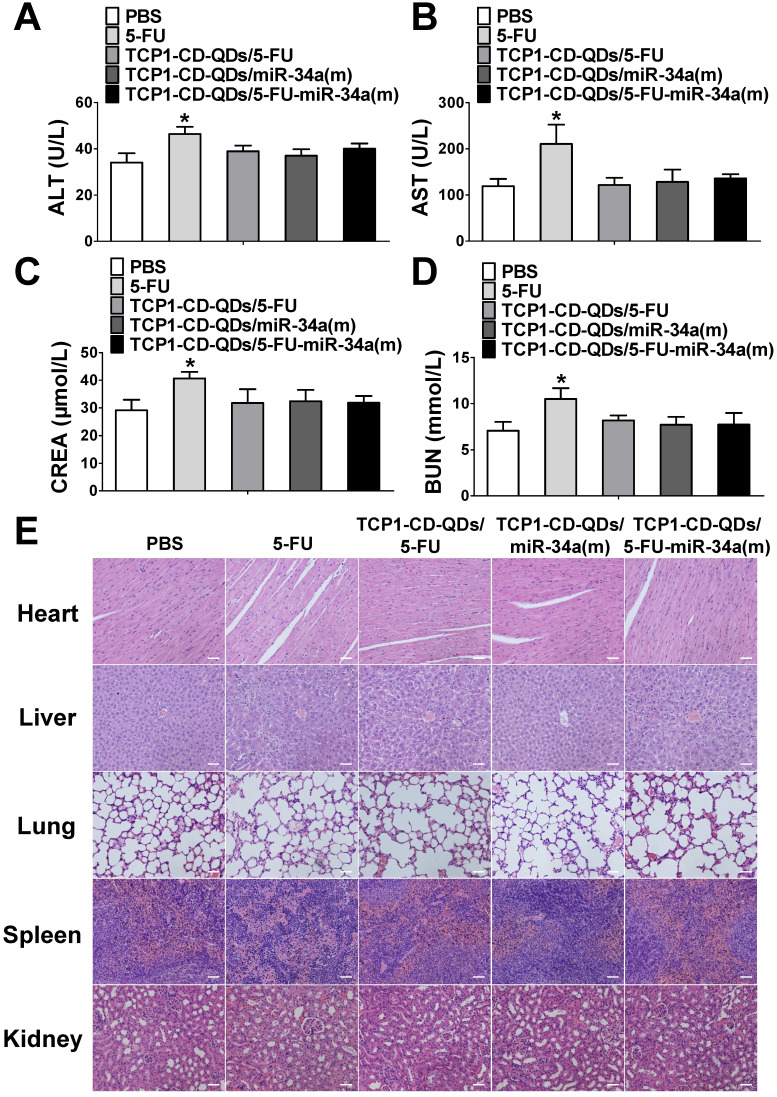
** Assessment of the systemic side effects of drugs in the different treatment groups. (A-D)** ALT, AST, CRE and BUN levels in serum were examined after treatment with PBS, free 5-FU, TCP1-CD-QDs/5-FU, TCP1-CD-QDs/miR-34a(m) and TCP1-CD-QDs/5-FU-miR-34a(m). **(E)** Effect of TCP1-CD-QD nanocarriers loaded with drugs on the histopathology of the major organs of treated CRC-bearing mice as shown by H&E staining. Tissues were collected from the heart, liver, lung, spleen and kidney. Bar: 50 µm.

Considering the side effects of chemotherapeutic drugs and nucleotide drugs 57, 58, we evaluated the potential biotoxicity of TCP1-CD-QD nanocarriers loaded with 5-FU and miR-34a(m) in major organs. First, several serum biochemical indicators related to liver and kidney functions 59 were examined after treatment with PBS, free 5-FU, TCP1-CD-QDs/5-FU, TCP1-CD-QDs/miR-34a(m) and TCP1-CD-QDs/5-FU-miR-34a(m). As shown in **Figure 8A-D**, treatment with TCP1-CD-QD nanocarriers loaded with drugs resulted in similar levels of alanine aminotransferase (ALT), aspartate aminotransferase (AST), creatinine (CRE) and blood urea nitrogen (BUN) compared to PBS treatment, while the levels in the free 5-FU-treated group were significantly higher than those in the other groups of tumor-bearing mice. At the same time, histopathological staining of the heart, liver, spleen, lung and kidney was also analyzed after treatment with PBS, free 5-FU, TCP1-CD-QDs/5-FU, TCP1-CD-QDs/miR-34a(m) and TCP1-CD-QDs/5-FU-miR-34a(m). No obvious morphological changes in the major organs were observed among these groups (**Figure 8E**). Biodegradability, biocompatibility and biosafety have always been the primary prerequisites for the biological application of QDs-based nanocarriers 60. Currently, the research conclusions on the biodegradability and biotoxicological effects of QDs were not uniform. The reason for this phenomenon is that the types of quantum dots used are different, and each quantum dot has unique physical and chemical characteristics and determines its potential biotoxicity, such as particle size, shape, surface charge, and surface modification and so on 61, 62. Early studies have shown that bare-core QDs can degrade and release Cd^2+^, resulting in toxic effects 26, 63. Therefore, in order to reduce the toxic effect of QDs due to degradation and release, and to enhance the stability of QDs, this study used the Cd and Te as core and Zn and S as shell. Some studies have been shown that in the case of a ZnS shell, the Cd^2+^ released by quantum dots is almost negligible 64. In addition, the surface-modified QDs can be made into hydrophilic particles. Some studies have been shown that different surface characteristics may lead to different biological degradation effects or biotoxicity 65, 66. In this study, the release of toxic molecules of QDs can be minimized by modification of CDs and the binding of peptides.

The biodistribution of agents directly reflects their possible toxicity on organs and antitumor effects *in vivo*. To investigate the tissue distribution of TCP1-CD-QD nanocarriers, mice bearing tumors derived from DLD1 cells line was administered with QDs and TCP1-CD-QD nanocarriers intravenously. The nude mice were dissected at 24 h to determine the fluorescence intensity of their isolated organs, including their heart, liver, lung, spleen, kidney, as well as that of the tumor (**Figure S11**). Compared to the QDs group, strong fluorescent signals were detected in area of tumors on 24 h after TCP1-CD-QDs nanocarriers injection, suggesting that could TCP1-CD-QDs nanocarriers could targeting accumulate within tumor tissue. Weak fluorescent signals were also observed in the liver and kidney, but not in other organs, indicating that TCP1-CD-QDs nanocarriers displayed a superior biodistribution pattern. These results indicate that our nanocarrier-based co-delivery system has undetectable side effects due to its tumor-targeted effects.

## Conclusion

Here, we designed novel CRC-targeting nanocarriers based on QDs through self-assembly driven by host-guest interactions. Our TCP1-CD-QD nanocarriers can simultaneously deliver the chemotherapeutic drug 5-FU and nucleotide drug miRNA-34a mimics targeted to CRC cells for use in combination therapy. Our results showed that the co-delivery of 5-FU and miR-34a(m) not only substantially enhanced the anti-CRC activity of 5-FU by silencing sirt1 expression but also suppressed CRC cell migration by targeting CD44, suggesting that co-delivery of 5-FU and miR-34a(m) could achieve synergistic effects on tumor suppression with undetectable side effects *in vitro* and *in vivo*. Of note, CRC PDX models were used to verify the therapeutic effect of our nanoplatform. Our results showed that the use of TCP1-CD-QD nanocarriers loaded with 5-FU and miR-34a(m) could efficiently target CRC cancer therapy in high-fidelity, clinically relevant patient-derived tumor models. Altogether, the co-delivery system of chemotherapeutic drugs and nucleotide drugs based on nanocarriers offers a promising strategy for enhanced CRC targeted therapy.

## Supplementary Material

Supplementary figures and tables.

## Abbreviations

PDX: patient-derived tumor xenograft
QD: quantum dot
CD: β-cyclodextrin
ADA: adamantine
TCP1: TCP1 peptide
CRC: colorectal cancer
5-FU: 5-fluorouracil
miR-34a(m): microRNA-34a mimics
CdTe: cadmium telluride
ZnS: zinc sulfide
MPA: 3-mercaptopropyl acid
APBA: 3-aminophenyl boronic acid
PEG: polyethylene glycol
TEM: transmission electron microscopy
DLS: dynamic light scattering
NMR: Nuclear magnetic resonance
DMEM: Dulbecco's modified Eagle medium
FBS: fetal bovine serum
CLSM: confocal laser scanning microscopy
DAPI: 4',6-diamidino-2-phenylindole
LC: loading capacity
EE: encapsulation efficiency
H&E: hematoxylin and eosin
IHC: immunohistochemical
