# Co-delivery of 5-fluorouracil and miRNA-34a mimics by host-guest self-assembly nanocarriers for efficacious targeted therapy in colorectal cancer patient-derived tumor xenografts: Erratum

**DOI:** 10.7150/thno.76869

**Published:** 2022-08-18

**Authors:** Jianbin Xu, Guolin Zhang, Xin Luo, Di Wang, Wei Zhou, Yan Zhang, Wei Zhang, Jiaxin Chen, Qing Meng, Engeng Chen, Heng Chen, Zhangfa Song

**Affiliations:** 1 Department of Colorectal Surgery, Sir Run Run Shaw Hospital, School of Medicine, Zhejiang University, Hangzhou, Zhejiang, 310016, P.R. China.; 2 State Key Laboratory of Medicinal Chemical Biology, Nankai University, Tianjin, 300350, P.R. China.; 3 Shenzhen Key Laboratory of Polymer Science and Technology, Guangdong Research Center for Interfacial Engineering of Functional Materials, College of Materials Science and Engineering, Shenzhen University, Shenzhen 518060, P.R. China.

The authors apologize that the original supplementary material of the above article contains errors in Figures S5 and S7 due to cell line problems. Therefore, we would like to delete the Figures S5 and S7 in the Supplementary Material. The new supplementary material file is provided in the link below.

As a result of this change, in the PDF of the original paper (page 2482, left column), the phase “In addition, as shown in Figure S5 and Figure S6” should change to “In addition, as shown in Figure S6”, and the phase “As shown in Figure S7 and Figure S8” should change to “As shown in Figure S8”.

The authors confirm that these corrections do not change the result interpretation or conclusions of the article. The authors are deeply sorry and sincerely apologize for any inconvenience or misunderstanding that may have caused.

## Supplementary Material

Supplementary figures and tables.

