# Supplementary material for: Co-delivery of 5-fluorouracil and miRNA-34a mimics by host-guest self-assembly nanocarriers for efficacious targeted therapy in colorectal cancer patient-derived tumor xenografts
Source: Theranostics. 2021 Jan 1;11(5):2475–89. doi: 10.7150/thno.52076 (PMC7797688; doi:10.7150/thno.52076)
Supplement: Supplementary file 1 — Supplementary figures and tables. [file thnov11p2475s1.pdf]

Supplementary material

Title： Co-delivery of 5-fluorouracil and miRNA-34a mimics by host-guest self-assembly nanocarriers for efficacious targeted therapy in colorectal cancer patient-derived tumor xenografts

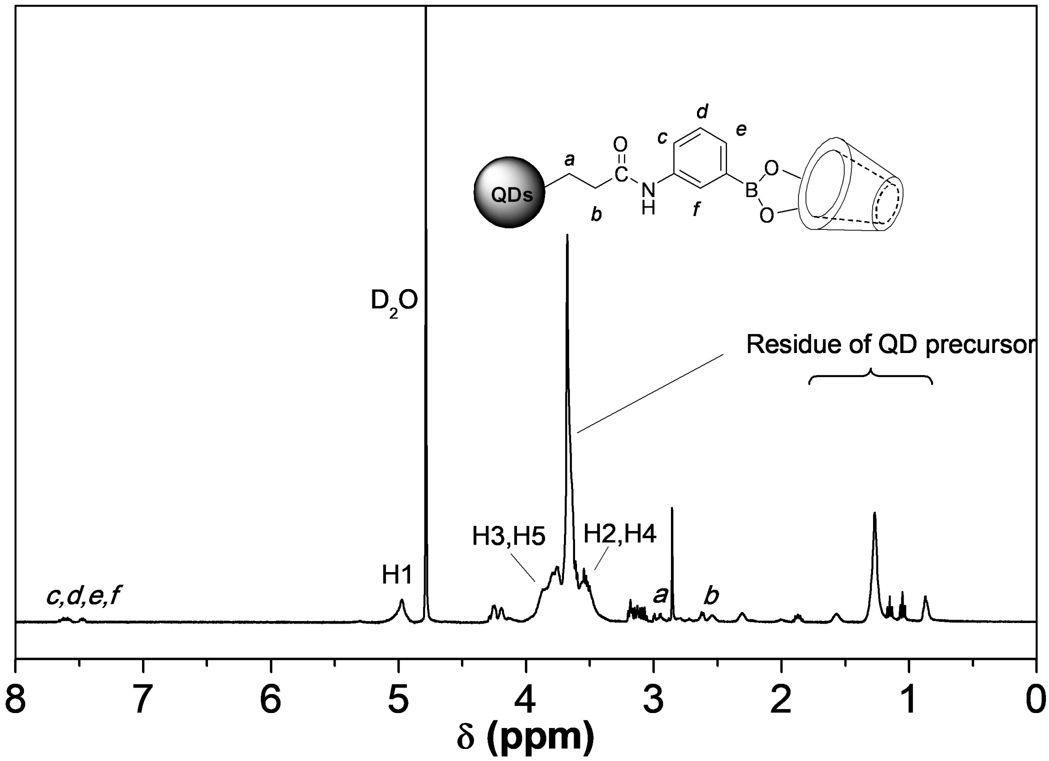

Figure S1. 1H NMR spectrum of CD-QDs in D2O.

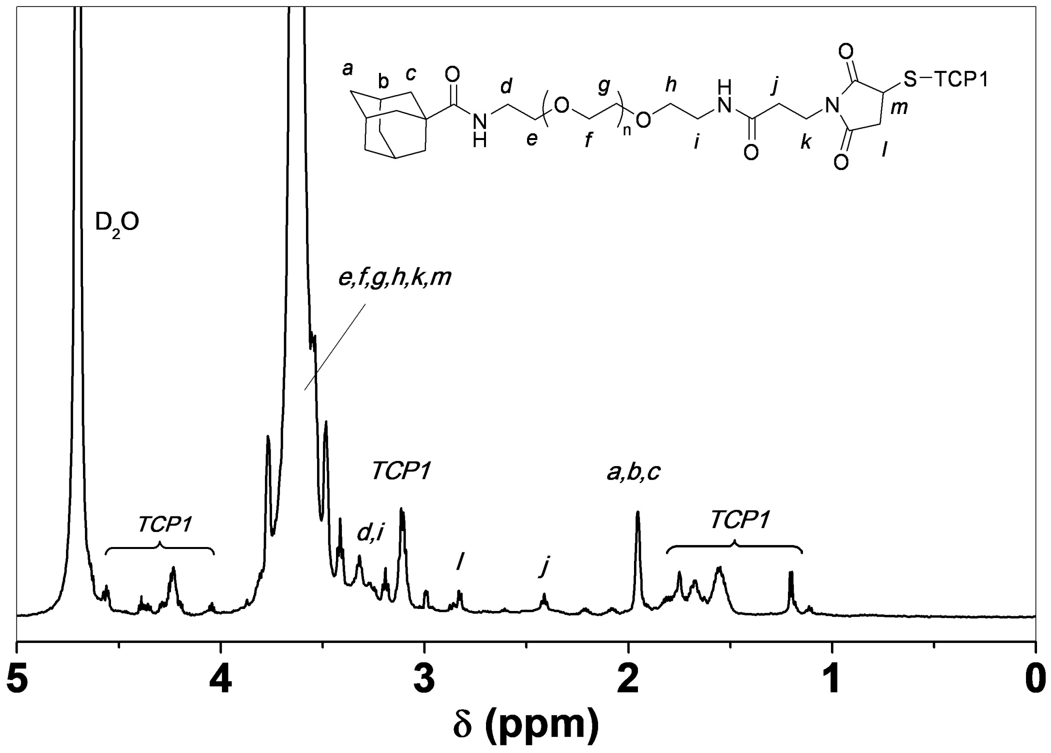

Figure S2. 1H NMR spectrum of ADA-PEG-TCP1 in D2O.

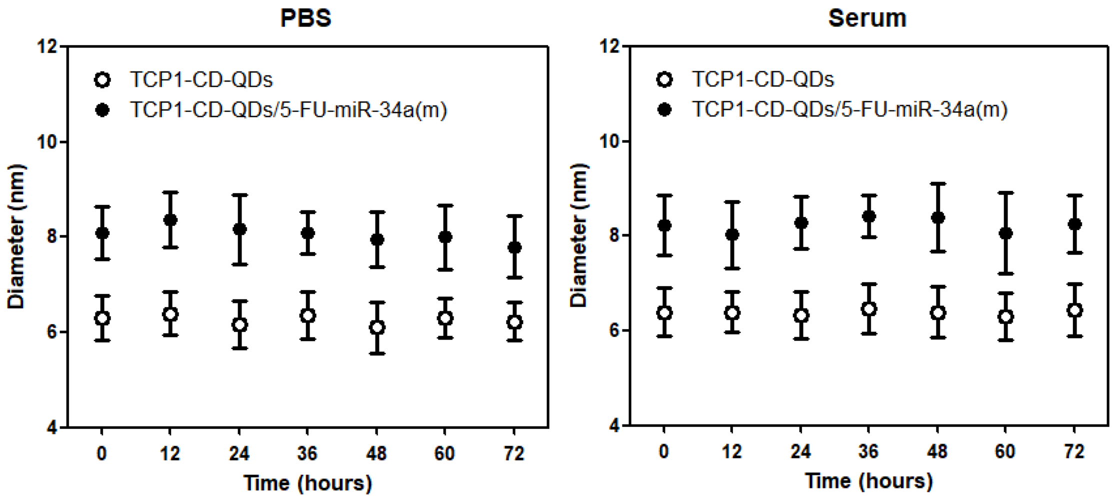

Figure S3. Stability of TCP1-CD-QDs and TCP1-CD-QDs/5-FU-miR-34a(m) in PBS and serum for 72 h.

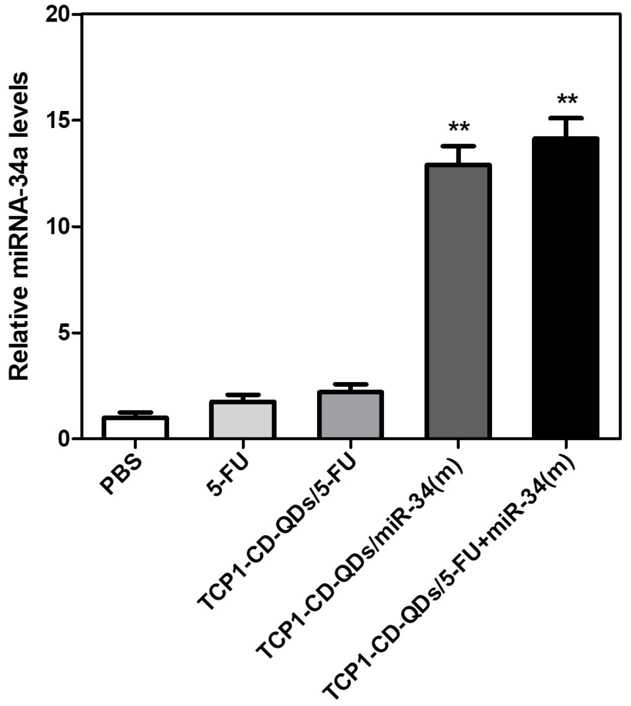

Figure S4. miR-34a expression in CRCs after delivery of miR-34a(m) by TCP1-CD-QD nanocarriers was measured by RT-PCR, and U6 small nuclear RNA was used as an internal control. The data are reported as the mean ± SD of the experiments (n=3).

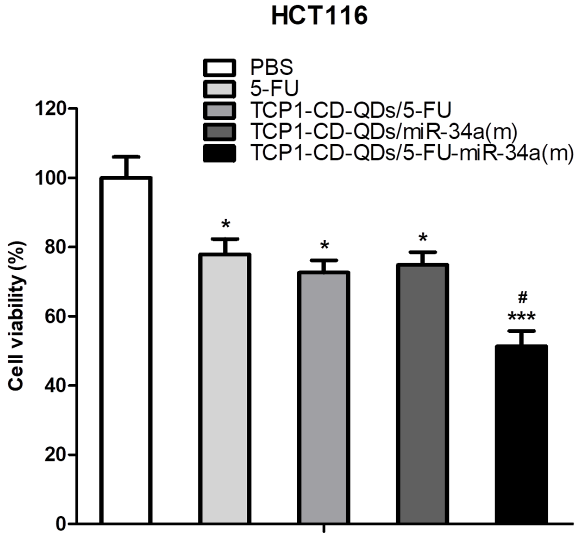

Figure S5. Cell viability of cells treated with PBS, free 5-FU, TCP1-β-CD-QDs/5-FU, TCP1-β-CD-QDs/miR-34(m) and TCP1-β-CD-QDs/5-FU+miR-34(m) for HCT116 cell line. The concentration of 5-FU at 2 μM and miR-34a(m) at 25 nM in all treatments for 48 h was measured.

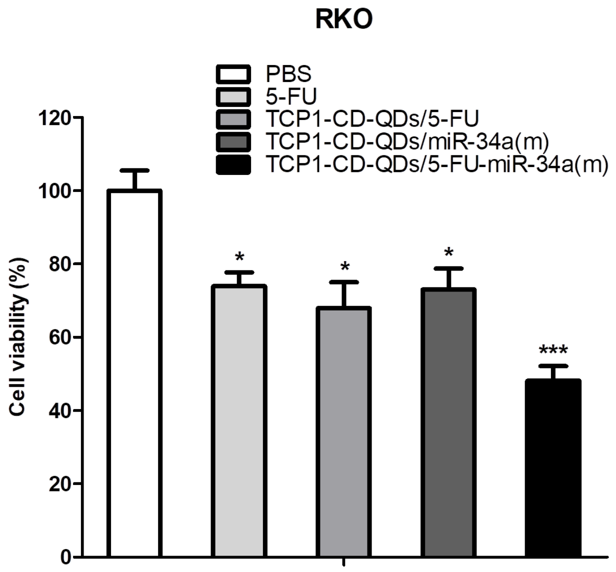

Figure S6. Cell viability of cells treated with PBS, free 5-FU, TCP1-β-CD-QDs/5-FU, TCP1-β-CD-QDs/miR-34(m) and TCP1-β-CD-QDs/5-FU+miR-34(m) for RKO cell line. The concentration of 5-FU at 2 μM and miR-34a(m) at 25 nM in all treatments for 48 h was measured.

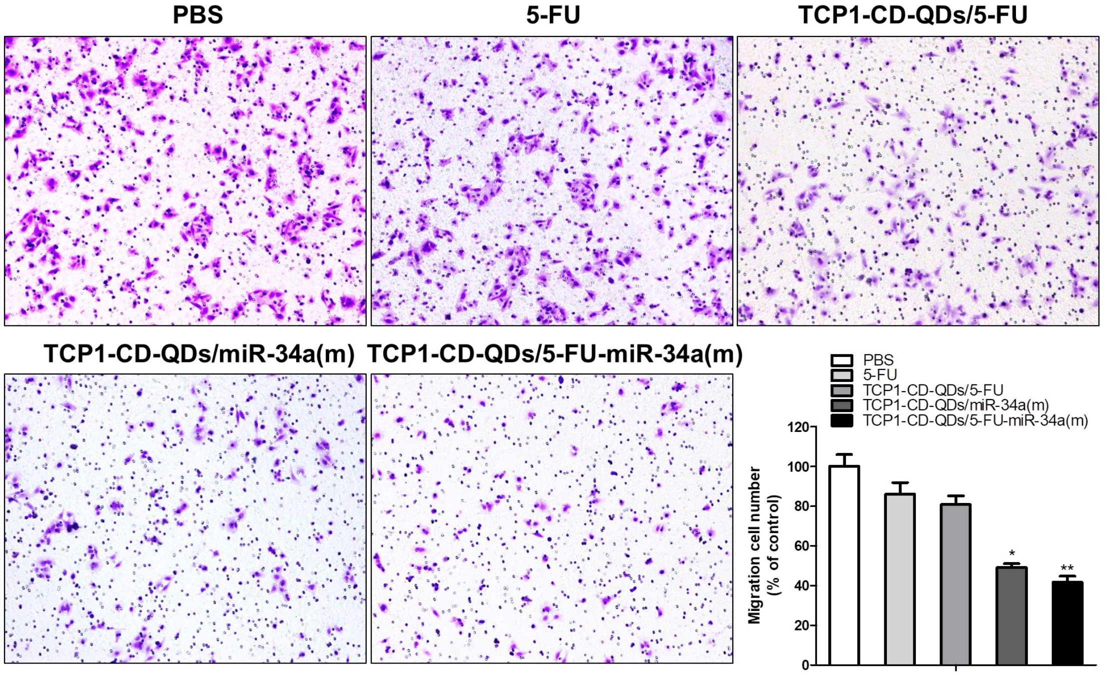

Figure S7. Transwell assay of treatments with PBS, free 5-FU, TCP1-CD-QDs/5-FU, TCP1-CD-QDs/miR-34(m) and TCP1-CD-QDs/5-FU+miR-34(m) for HCT116 cell line. The concentration of 5-FU at 2 μM miR-34a(m) at 25 nM in all treatments for 24 h.

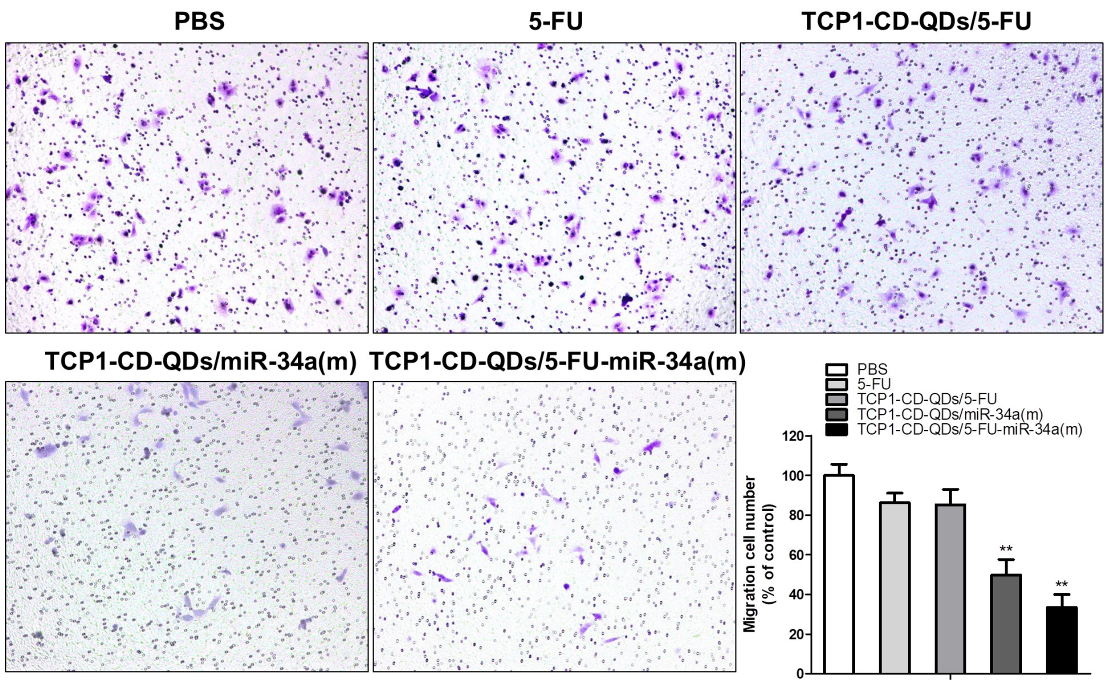

Figure S8. Transwell assay of treatments with PBS, free 5-FU, TCP1-CD-QDs/5-FU, TCP1-CD-QDs/miR-34(m) and TCP1-CD-QDs/5-FU+miR-34(m) for RKO cell line. The concentration of 5-FU at 2 μM miR-34a(m) at 25 nM in all treatments for 24 h.

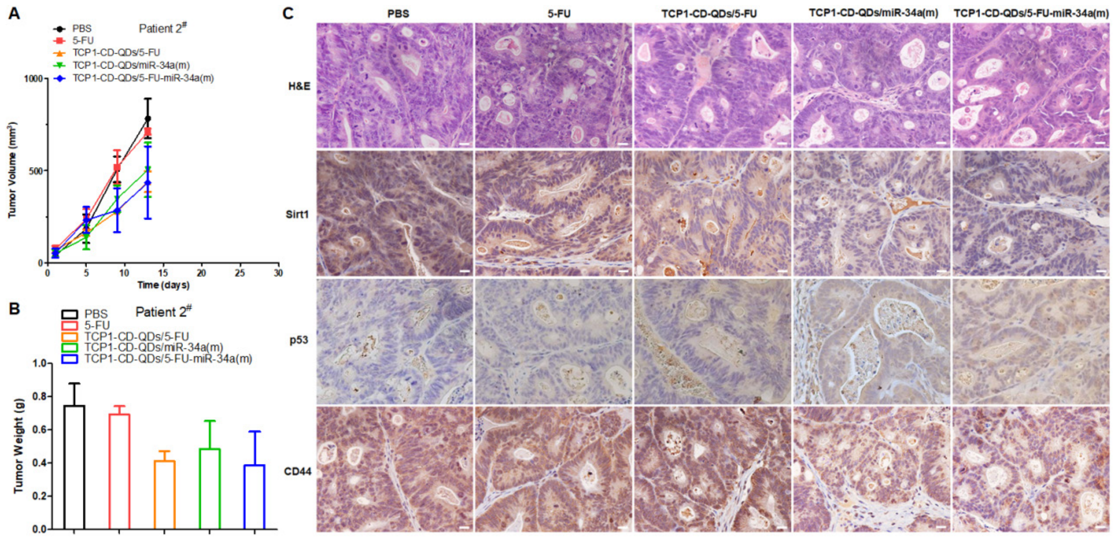

Figure S9. Suppression of subcutaneous tumor growth by TCP1-CD-QDs/5-FU+miR-34(m) in PDX models. The tumor models were treated with PBS, free 5-FU, TCP1-CD-QDs/5-FU, TCP1-CD-QDs/miR-34a(m) and TCP1-CD-QDs/5-FU-miR-34a(m) group, respectively. (A) The tumor growth in PDX model of treatments in 5 groups. (B) The tumor weight in PDX model of treatments in 5 groups. (C) Representative sirt1, p53 and CD44 immunohistochemistry images of treatments in 5 groups. Bar: 20 μm.

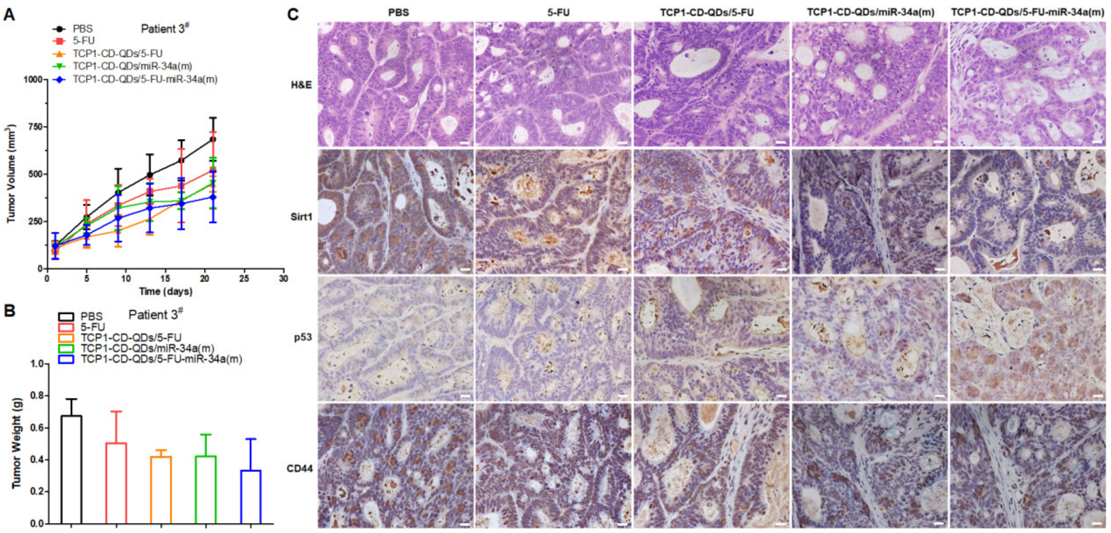

Figure S10. Suppression of subcutaneous tumor growth by TCP1-CD-QDs/5-FU+miR-34(m) in PDX models. The tumor models were treated with PBS, free 5-FU, TCP1-CD-QDs/5-FU, TCP1-CD-QDs/miR-34a(m) and TCP1-CD-QDs/5-FU-miR-34a(m) group, respectively. (A) The tumor growth in PDX model of treatments in 5 groups. (B) The tumor weight in PDX model of treatments in 5 groups. (C) Representative sirt1, p53 and CD44 immunohistochemistry images of treatments in 5 groups. Bar: 20 μm.

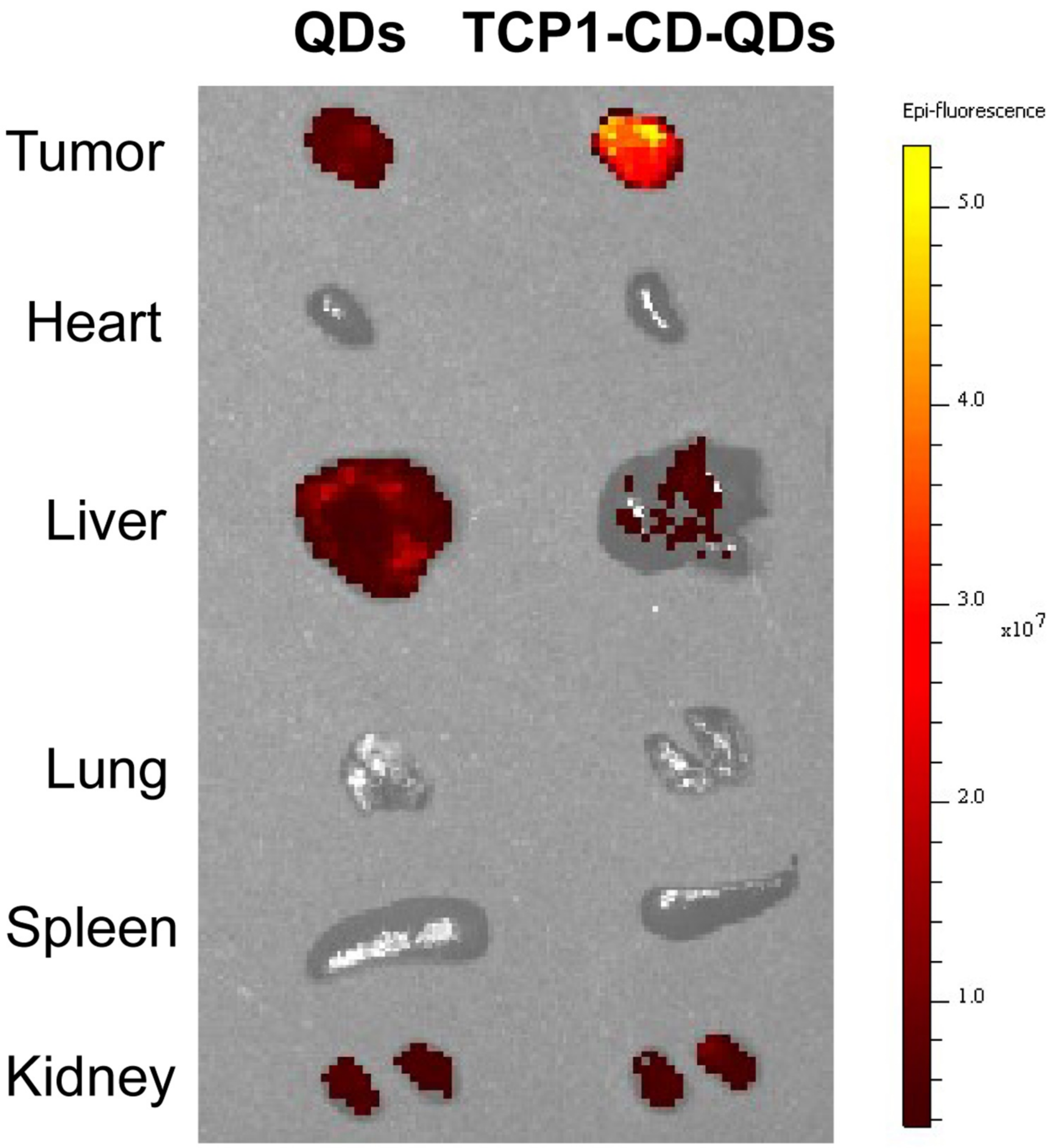

Figure S11. Biodistribution of QDs and TCP1-CD-QDs in tumor-bearing mice. The fluorescence imaging of tumors and major organs (heart, liver, lung, spleen, kidney) at 24 h after mice had been treated with QDs and TCP1-CD-QDs.

Table S1. DLS and Zeta potential of TCP1-CD-QDs and TCP1-CD-QDs/5-FU-miR-34a(m)

Nanocomplexes	TCP1-CD-QDs	TCP1-CD-QDs/5-FU-miR-34a(m)
DLS (nm)	6.3 ± 0.8	8.1 ± 1.0
Zeta potential (mV)	18.3 ± 0.8	10.5 ± 0.9
